# Development of Chitosan-Coated Liposomes for Oral Delivery of Nadolol: Preparation, Characterization, and *in vitro* Permeability Studies

**DOI:** 10.2174/0113816128401910250706133608

**Published:** 2025-07-16

**Authors:** Esra İpekci, Emre Şefik Çağlar, Mustafa Sinan Kaynak, Evren Gündoğdu, Neslihan Üstündağ Okur

**Affiliations:** 1 Department of Pharmaceutical Technology, Faculty of Pharmacy, University of Health Sciences, İstanbul, 34000, Turkey;; 2 Department of Pharmaceutical Biotechnology Technology, Faculty of Pharmacy, University of Health Sciences, İstanbul, 34000, Turkey;; 3 Department of Pharmaceutical Technology, Faculty of Pharmacy, Anadolu University, Eskişehir, 26000, Turkey;; 4 Department of Radiopharmacy, Faculty of Pharmacy, Ege University, İzmir, 35000, Turkey

**Keywords:** Nadolol, liposomes, chitosan, permeability, hypertension, nanocarrier systems

## Abstract

**Introduction:**

This study aims to enhance the oral bioavailability of Nadolol (NDL), a β-blocker used in the management of hypertension, by incorporating it into a liposome-based delivery system. To improve the formulation’s stability, mucoadhesion, and permeability, chitosan coating was applied.

**Methods:**

Liposomes were prepared *via* the ethanol injection method using soy phosphatidylcholine and diacetyl phosphate. Chitosan coating was applied by adding chitosan solution (1% *v/v* acetic acid) at different chitosan-to-lipid ratios (0.1-0.4 *w/w*). The optimal formulation was selected based on particle size, PDI, and zeta potential. Characterization included encapsulation efficiency, drug loading, enzymatic stability, drug release, and Caco-2-based cytotoxicity and permeability assays.

**Results:**

The particle size and polydispersity index of the optimized formulations, L1-NDL, L2-NDL, L1C-NDL, and L2C-NDL, were measured as 27.02 ± 0.18 nm, 24.55 ± 0.22 nm, 160.10 ± 3.17 nm, 161.00 ± 2.30 nm, 0.39 ± 0.01, 0.37 ± 0.01, 0.19 ± 0.01, and 0.18 ± 0.02. Encapsulation efficiencies of 56.01 ± 3.70% and 43.87 ± 1.24% were recorded for L1C-NDL and L2C-NDL, respectively, while drug loading capacities were 61.47 ± 2.03% and 67.80 ± 0.74%, respectively. In an enzymatic degradation study, it was found that chitosan coating increased the stability of liposomes in the gastric media. The *in vitro* release was higher at both pH 1.2 and 6.8. Caco-2 assays confirmed >95% cell viability and enhanced permeability in the apical-to-basolateral direction. In the permeability study, chitosan-coated liposomal formulations demonstrated enhanced transport in the apical-to-basolateral direction, indicating improved intestinal permeability.

**Conclusion:**

Chitosan-coated liposomes improved NDL’s stability and permeability, showing promise as an effective oral delivery system.

## 1 INTRODUCTION

Nadolol (NDL) is an antihypertensive agent belonging to the beta-blocker class, which is mainly used in the treatment of hypertension and angina [1, 2]. It shows its antihypertensive effect by reducing peripheral vascular resistance [3]. Hypertension is among diseases that affect up to billions of people in society and have a significant morbidity-mortality rate [4-6]. It is a cardiovascular disease that often occurs without a specific cause, characterized by blood pressure above 140/90 [7, 8]. Drugs that are routinely used in the treatment of hypertension directly or indirectly reduce vascular resistance, regulate cardiac contractility, or exert diuretic effects through various mechanisms [9]. Although NDL is clinically approved by the Food and Drug Administration (FDA) for the treatment of angina and hypertension, it is also prescribed for migraine prophylaxis, atrial fibrillation, and ventricular arrhythmia [3, 9]. Despite its clinical efficacy, NDL suffers from poor oral bioavailability mainly due to its limited membrane permeability and low intestinal absorption. NDL is a Biopharmaceutics Classification System Class III drug, characterized by high solubility but low permeability, which presents significant challenges in achieving effective systemic drug concentrations through oral administration [10, 11]. Its oral bioavailability is around 35% [10].

To address the challenges of delivering low-permeability drugs like NDL, researchers have explored several formulation approaches. These include the use of absorption enhancers, solid lipid nanoparticles, polymer-based systems, and various vesicular carriers, such as niosomes, ethosomes, and liposomes [12]. Among these, liposomes have emerged as promising carriers due to their ability to encapsulate both hydrophilic and lipophilic drugs, improve drug solubility, and enhance membrane interaction and transport [13-15]. Discovered by Bangham in the 1960s, liposomes are noted for being biocompatible, biodegradable, and exhibiting low immunogenicity and toxicity [16-18]. Liposomes can also be adapted for various application routes, such as oral, transdermal, ocular, parenteral, and pulmonary, and can be used in both diagnosis and treatment in the clinic [14, 19].

In this study, we prepared chitosan-coated liposomes using the ethanol injection method to improve the oral delivery of NDL. This approach was designed to enhance drug stability in the gastrointestinal tract, prolong mucosal residence time through mucoadhesion, and ultimately increase oral bioavailability. Although the oral route is one of the most preferred by patients, clinicians, and the pharmaceutical industry due to its convenience and cost-effectiveness, it often results in significantly reduced bioavailability due to the harsh conditions within the gastrointestinal tract [11, 20]. Considering these limitations, various drug delivery strategies and modified formulations have been developed. In this context, liposomes have gained considerable interest as promising carriers for oral drug administration [21, 22].

Liposomes are mainly composed of phospholipid bilayers, which offer protection to the encapsulated drug against the harsh environment of the gastrointestinal tract, including acidic pH and enzymatic activity [17, 23, 24]. However, when administered orally, conventional liposomes can encounter issues such as instability in acidic gastric conditions, early drug release, and limited interaction with the mucosal surface [13, 25, 26]. These drug delivery systems are open to developing drug delivery systems as they are compatible with modifications such as targeting *via* ligands, including polymers, vitamins, peptides, and carbohydrates, prolonging residence time in the bloodstream through PEGylation, and controlled release [16, 17]. In this study, to overcome these disadvantages, the liposome surface was modified with mucoadhesive polymers, such as chitosan. Chitosan is a natural polysaccharide obtained by deacetylation of chitin. Chitosan is a naturally derived, positively charged polysaccharide known for its strong mucoadhesive properties. It can also temporarily open tight junctions between epithelial cells, enhancing drug transport through the paracellular route [27-29]. While the chitosan coating facilitates electrostatic bonding to the membrane due to its cationic charge, it opens epithelial cell tight junctions and provides an extended retention time *via* its bioadhesive feature, consequently contributing to an increase in bioavailability [30-32]. Additionally, chitosan is antiviral, antibacterial, hemostatic, non-toxic, biocompatible, and biodegradable [33-35].

In this study, novel chitosan-coated liposomal formulations loaded with NDL were successfully developed with the primary objective of enhancing mucoadhesive properties and improving the oral bioavailability of the drug. The rationale behind this approach lies in the ability of chitosan to interact with mucosal surfaces, thereby extending the residence time of the liposomes in the gastrointestinal tract and potentially facilitating more efficient drug absorption. To thoroughly evaluate the potential of these novel nanocarrier systems for oral drug delivery, a comprehensive set of studies was conducted. These included detailed physicochemical characterization of the formulations to assess parameters, such as particle size, surface charge, morphology, and encapsulation efficiency. In addition, *in vitro* drug release studies were performed to investigate the release kinetics of NDL from both coated and uncoated liposomes under simulated gastrointestinal conditions. The stability of the liposomes in the presence of digestive enzymes was also evaluated through enzymatic degradation studies, providing insight into the protective effect of the liposomal and chitosan layers against harsh gastrointestinal tract conditions. Furthermore, cytotoxicity studies were carried out using relevant cell lines to determine the biocompatibility and safety of the developed formulations. Lastly, *in vitro* permeability studies, often using human colon epithelial cancer cell line (Caco-2) cell models, were employed to assess the transport potential of the formulations across intestinal epithelial barriers. Collectively, these experiments aimed to establish a solid foundation for the use of chitosan-coated liposomes as a promising platform for the effective and safe oral delivery of NDL.

## 2 MATERIALS AND METHODS

### 2.1 Materials

NDL was purchased from ChemCruz (USA). L-α-phosphatidylcholine (SPC), Cholesterol (CH), and Dicetyl Phosphate (DCP) were purchased from Avanti Polar Lipids (USA). Sodium chloride was obtained from ISOLAB chemicals (Germany). Low molecular weight chitosan (20-200 cps), Cinnamon Oil (CO), methanol, sodium hydroxide, pepsin, and pancreatin were purchased from Sigma (Germany). Hydrochloric acid and acetic acid were from Merck (Germany). Dulbecco's Modified Eagle Medium was purchased from Gibco (England). All other reagents and buffer solution components were of analytical grade.

### 2.2 Preparation of Liposomes

Liposomes were prepared *via* the ethanol injection method with some modifications. Briefly, the lipid mixture L1 (SPC:DCP:CH (mM) 5:2:1, 0.38% CO) and L2 (SPC:DCP:CH (mM) 3:0.5:1) were dissolved in ethanol (Table **1**). NDL was dissolved in ethanol and added to the lipid phase. NDL-loaded L1 and L2 formulations were coded as L1-NDL and L2-NDL. The lipid mixture was added dropwise to 5 mL of distilled water at 1000 rpm on a magnetic stirrer. The solution was stirred at 1000 rpm and 80℃ for 210 minutes in a magnetic stirrer until the ethanol had evaporated [36, 37].

For the chitosan solution, it was dissolved in a 1% acetic acid solution at a concentration of 1% and filtered through a 0.45 µm membrane filter [38]. It was completed with distilled water to the amount determined at the optimum rate. The prepared liposomes were added dropwise to the chitosan solution and mixed in a magnetic stirrer at 850 rpm for 60 minutes. Formulations L1 and L2 were coated with chitosan and designated as L1C and L2C. NDL-loaded chitosan-coated formulations were referred to as L1C-NDL and L2C-NDL [39].

### 2.3 High Performance Liquid Chromatography (HPLC) Analysis

HPLC (Agilent 1200) conditions with a UV detector were studied at 206 nm using a C18 analytical column (Phenomenex, 5 µm, 250 x 4.6 mm), a flow rate of 1 mL/min, and an injection volume of 20 µL. The mobile phase was prepared by adding 1 mL of hydrochloric acid to a mixture of 0.293% sodium chloride and methanol (65:35). The guidelines of the International Council for Harmonisation of Technical Requirements for Pharmaceuticals for Human Use were followed for analytical method validation [40].

### 2.4 Liposome Particle Size Distribution and Zeta Potential Determination

Particle size, PDI, and zeta potential were measured by dynamic light scattering using a zetasizer (Malvern Zetasizer Ultra, Malvern Instruments). The droplet size and PDI were measured using disposable cells at an angle of 173° at 25°C. Zeta potential was measured using zeta cells at 25°C. Experiments were performed in triplicate [41].

### 2.5 Encapsulation Efficiency

The dialysis membrane containing 1 mL of liposomes and chitosan-coated liposomes was placed in 9 mL of distilled water and centrifuged at 5000 rpm, 4℃ for one hour (Sigma 3-18 KS). The aqueous fraction was taken and analyzed by HPLC. Experiments were performed in triplicate [42].

Encapsulation efficiency was calculated by Equation 1 below.


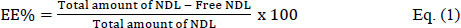


### 2.6 Drug Loading

The lipids in the formulation were dissolved in absolute ethanol to form the lipid phase of the liposome. NDL was also dissolved in the same ethanolic lipid solution to ensure homogeneous distribution of the drug. Following dissolution, liposomes were prepared as given in section 2.2. After the encapsulation efficiency study, the non-chitosan-coated liposomes remaining in the membrane were left in 9 mL of methanol. Chitosan-coated liposomes were deposited in 9 mL of 1% acetic acid solution and determined by HPLC. Experiments were performed in triplicate [42].

Drug loading was calculated by Equation 2 below:


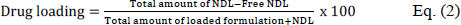


### 2.7 Enzymatic Degradation Studies

A solution was prepared by dissolving 0.2 grams of sodium chloride and 0.32 grams of pepsin in 0.7 mL of concentrated hydrochloric acid, and the volume was adjusted to 100 mL with distilled water. Hydrochloric acid was adjusted to pH 1.2. The prepared solution was equilibrated at 37 ± 0.5°C prior to the experiment [42].

Formulation (1 mL) was added to 9 mL of enzyme-containing medium. At 30, 60, and 120 minutes, samples were taken from the environment and centrifuged at 3500 rpm for 10 minutes. Samples were taken from the supernatant. Particle size, PDI, and zeta potential values were measured with a zetasizer [42].

0.68 grams of monopotassium phosphate was dissolved in 25 mL of distilled water, 7.7 mL of 0.2 M sodium hydroxide was added, and the volume was adjusted to 50 mL with distilled water. Then, 1 gram of pancreatin was added, and the volume was made up to 100 mL with distilled water. The pH setting was adjusted to 6.8 with 1 N sodium hydroxide. Following this, the prepared solution was equilibrated at 37 ± 0.5°C prior to the experiment [42].

Formulation (1 mL) was added to 9 mL of enzyme-containing medium. At 30, 60, 120, 240, and 360 minutes, samples were taken from the environment and centrifuged at 3500 rpm for 10 minutes. Samples were taken from the supernatant. Particle size, PDI, and zeta potential values were measured with a zetasizer. The enzymatic degradation study experiment was performed in triplicate [42].

### 2.8 *In Vitro* Release Studies

The release study was performed using the dialysis bag method (Spectra/Por Dialysis membrane/molecular weight of 12-14 kDa). Gastric (pH 1.2) and intestinal (pH 6.8) media were used as the release medium. 25 mL chitosan-coated liposomes (1.6 mg/mL) formulations were placed in the dialysis bag and closed with closures. The filled dialysis bags were immersed in 375 mL of gastric and intestinal media, providing sink conditions, and mixed at 37 ± 0.5°C with a magnetic stirrer at 50 rpm. The membrane was held in saline before use. In the gastric medium, 500 µl samples were taken at 15, 30, 45, and 60 minutes, followed by 2, 3, 4, 6, and 8 hours; in the intestinal medium, samples were collected at 15, 30, 45, and 60 minutes, followed by 2, 3, 4, 6, 8, 10, 12, and 24 hours, and quantified using HPLC. The system was completed with the same media and amount in each sample collection at 37 ± 0.5℃. Three parallel release studies were conducted for each formulation [43].

The *in vitro* release study calculations were performed with the equation given in Equation 3.


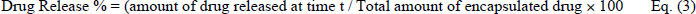


### 2.9 Cell Culture Studies

The formulations were studied with the Caco-2 cell line for *in vivo* evaluation. The cell lines were purchased from Ministry of Agriculture and Foresty-Sap Institute, Republic of Türkiye (https://vetkontrol.tarimorman.gov.tr/sap/Sayfalar/EN/Anasayfa.aspx). They were seeded at a density of 1 x 10^5^ cells in a six-well membrane support system (Transwell^®^) at 37°C under 90% humidity atmospheric conditions with 5% CO_2_. Cells were cultured in Dulbecco's Modified Eagle Media (DMEM) for 21 days under appropriate conditions. The media was changed every two days. The passage numbers of the cells were 21-55. Transepithelial Electrical Resistance (TEER) was measured to detect each cell monolayer. TEER measurement was made with epithelial voltammetry before and after the experiment [44].

Cell viability was evaluated by MTT (3-[4,5-dimethylthiazol-2-yl]-2,5-diphenyltetrazolium bromide) assay. MTT is a yellow-colored formazan salt. The test is based on the mitochondrial dehydrogenase enzyme in dividing cells, which converts MTT into purple-colored formazan crystals [45]. The IC_50_ values of the formulations were calculated using the GraphPad Prism Program.

Cell permeability evaluation was performed from apical to basolateral (A→B) and basolateral to apical (B→A). Formulations were added to the apical portion at 2.2 mL and 3.2 mL to the basolateral portion. For permeability analysis, samples were shaken at 37°C at 50 rpm for 2 hours. The samples were taken from the acceptor phase and were measured using HPLC. Three parallel release studies were conducted for each formulation [44].

### 2.10 Stability Studies

Particle size, PDI, zeta potential, mobility, and conductivity changes of the liposome formulations were studied at 5 ± 3°C under refrigerator conditions, and active ingredient quantification at 5 ± 3°C, 25 ± 2°C, 60 ± 5%, 40 ± 2°C, and 75 ± 5% relative humidity at initial, 3^rd^, 6^th^, 9^th^, and 12^th^ months. Three parallel release studies were conducted for each formulation [46-49]. The results of the stability studies were statistically analyzed using GraphPad Prism 8.0 (GraphPad Software, La Jolla, CA, USA). *p* < 0.05 was considered significant. A one-way analysis of variance test was used to compare the groups.

### 2.11 Statistical Analysis

The results were statistically evaluated using GraphPad Prism 8.0 (GraphPad Software, La Jolla, CA, USA). *p* < 0.05 was considered significant. A one-way analysis of variance test was used to compare the groups.

## 3 RESULTS AND DISCUSSION

### 3.1 Preparation and Characterization of Liposomes

In the present study, lipid concentrations were optimized through a trial-and-error approach based on the evaluation of particle size and Polydispersity Index (PDI). Various lipid ratios were tested, and the final composition was selected by considering the smallest particle size with the lowest PDI, reflecting uniform distribution and colloidal stability. The components of the optimally selected L1 and L2 liposomes are SPC, DCP, CH, and CO for L1 and SPC, CH, and DCP for L2, respectively. The components used in the formulations affect the characteristic properties of the liposome. SPC was selected as the primary phospholipid due to its biocompatibility and proven effectiveness in forming stable liposomal vesicles. SPC, derived from soybean phospholipids, is an unsaturated natural phospholipid that imparts high permeability to liposomes and influences their surface charge through its phosphate groups [16, 50]. CH was incorporated into the formulation to enhance the stability of the liposomes by intercalating within the bilayer membrane and altering membrane fluidity. Furthermore, CH has been reported to prolong the circulation time of liposomes from minutes to hours by reducing interactions with blood proteins [23]. A negatively charged lipid was used to strengthen electrostatic interactions with positively charged polymers, such as chitosan, and to improve the overall stability of the vesicles. By imparting a negative surface charge to the liposomes, DCP facilitated effective chitosan coating and contributed to the structural integrity of the delivery system [51, 52]. In one of the formulations, CO was incorporated as a functional excipient due to its multifaceted biological properties. Studies have shown that oils like CO can act as natural permeation enhancers by modulating lipid membrane fluidity, potentially improving the absorption of encapsulated drugs. Moreover, its antioxidant and antimicrobial properties may contribute to enhancing the physicochemical stability of liposomal formulations and extending shelf life. The inclusion of CO also allowed us to compare its impact on the physicochemical characteristics and performance of the liposomal system, providing a broader evaluation of formulation strategies [53].

Chitosan, a natural polysaccharide, is derived from chitin and is known for its biocompatible, biodegradable, mucoadhesive, and low-toxicity properties [54, 55]. Chitosan increases residence time and adsorption in the mucosal area, resulting in increased bioavailability [51]. It has been reported in literature that it inhibits the efflux pump, which reduces drug bioavailability and increases drug absorption by opening tight junctions [17, 31]. Furthermore, low molecular weight chitosan, which can be classified according to its acetylation degree, shows stronger mucoadhesive properties [25]. The free amino groups in the chitosan structure are protonated in acidic media and acquire a positive charge. The positive charge density of chitosan allows liposomes to be coated by creating an electrostatic interaction with anionic charges [56, 57]. In the literature, it has been stated that the coating of liposomes with chitosan is formed by forces such as Van der Waals, hydrogen bonding, and electrostatic interactions. The dominant force compared to others is thought to be the interaction between the positively charged amine groups (NH^3+^) of chitosan and the negatively charged phosphate groups (PO_4_^3-^) of the liposome [56]. Chitosan forms a positively charged layer on the vesicle surface by electrostatically binding to negatively charged liposomes. The positive charge of chitosan ensures the affinity of the liposomes to the negatively charged cell membrane [58].

The determination of the chitosan coating concentration is a critical step, as insufficient coating below the saturation concentration leads to inadequate surface coverage, while exceeding this concentration may result in flocculation and coagulation. To identify the optimal chitosan-to-lipid ratio, selected liposomes were coated with varying amounts of chitosan [56]. The coating was tested with concentrations in the range of 0.1, 0.2, 0.25, 0.3, and 0.4 (*w/w*) (Table **2**) and the optimum concentration was determined to be 0.25 (*w/w*) by evaluating the particle size, PDI, and zeta potential [59]. Chitosan was incorporated at a chitosan-to-lipid ratio of 0.25 (*w/w*) to form a surface coating aimed at enhancing mucoadhesiveness and stability. The occurrence of coating was confirmed by observing increases in particle size and PDI, as well as a distinct shift in zeta potential from negative to positive values. These changes indicate successful adsorption of the cationic chitosan polymer onto the negatively charged liposomal surface. This formulation strategy reflects established approaches in literature and supports the intended functionality of the delivery system. Liu *et al*. developed paclitaxel-loaded chitosan thioglycolic acid-coated liposomes for oral cancer therapy. In their study, liposomes were coated with chitosan at weight ratios between 0.0-0.4%. It was reported that there was no significant change in zeta potential after 0.3%, and this was considered the optimal ratio [60]. Similarly, in this study, no significant change in zeta potential was observed in coated liposomes after 0.25% (*w/w*) chitosan coating (Table **2**). Nguyen *et al*. loaded Berberine Hydrochloride (BH) into liposomes to overcome its low oral bioavailability and potential parenteral side effects. Liposomes were prepared using a lecithin-CH mixture and coated with CH. The zeta potential of uncoated liposomes was -39.5 ± 1.2 mV, while liposomes coated with 0.1% chitosan showed a zeta potential of 24.1 ± 0.5 mV [43]. Similarly, in this study, liposomes with a negative surface charge became positively charged after chitosan coating.

The particle sizes of uncoated liposomes for the L1 and L2 formulations were measured as 246.60 ± 5.09 nm and 350.90 ± 2.95 nm, respectively (Table **3**). Loading with NDL reduced the particle size of liposomes, with L1-NDL and L2-NDL formulations showing sizes of 27.02 ± 0.18 nm and 24.55 ± 0.22 nm, respectively (Table **3**).

Coating NDL-loaded liposomes with chitosan resulted in an increase in particle size (Table **3**). Similarly, in Lu *et al*.'s study on actoside-loaded chitosan-coated liposomes, particle size increased upon chitosan coating [39]. The particle sizes of L1C-NDL and L2C-NDL formulations were measured as 160.10 ± 3.17 nm and 161.00 ± 2.30 nm, respectively (Table **3**). The literature indicates that a particle size range of 50 to 200 nm is preferred for liposomes designed for drug delivery applications [61]. Dadashzadeh *et al*. noted that achieving the optimal saturation concentration of chitosan for liposome coating ensures a homogeneous coating across the liposome surface, leading to a more uniform size distribution and a lower PDI value [62]. Consistent with the literature, this study observed a decrease in PDI values with the optimal chitosan ratio (Table **3**). The NDL-loaded liposome formulations developed in this study exhibited PDI values below 0.5 (Table **3**), which is considered homogeneous in many studies [63].

One of the indicators of stability in colloidal systems is a high zeta potential, which prevents aggregation and flocculation through electrostatic repulsion [64-66]. Before chitosan coating, the negative zeta potential of the liposomes was attributed to the phosphate and carboxylic groups in phospholipids, as well as the presence of DCP [36]. The zeta potentials of L1-NDL and L2-NDL formulations were measured as -42.09 ± 0.29 mV and -50.91 ± 0.75 mV, respectively (Table **3**). After chitosan coating, the zeta potentials increased to 40.77 ± 2.17 mV and 37.84 ± 5.51 mV, respectively (Table **3**). These results clearly demonstrate that the positively charged amino groups of chitosan converted the surface charge of liposomes from negative to positive.

To determine the ideal coated formulation, the particle size and PDI value were considered as the lowest, while the zeta potential was considered as the highest. Accordingly, in both formulations, the liposomes coated with 0.25% chitosan concentration were identified as the optimal formulations.

### 3.2 Encapsulation Efficiency and Drug Loading

The encapsulation efficiency of chitosan-coated liposomes demonstrated a significant decrease compared to uncoated formulations. Specifically, the encapsulation efficiencies of L1C-NDL and L2C-NDL formulations were measured as 56.01 ± 3.70% and 43.87 ± 1.24%, respectively. Chitosan coating increased the difference in encapsulation efficiency between these formulations from 3% to 13%. Among all formulations, L1-NDL and L1C-NDL exhibited higher encapsulation efficiencies. While chitosan coating enhanced the drug loading capacity, it simultaneously reduced the encapsulation efficiency.

This trend is consistent with findings reported by Nguyen *et al*., who investigated berberine hydrochloride-loaded chitosan-coated liposomes. In their study, encapsulation efficiency decreased from 83.2 ± 0.4% to 78.4 ± 0.5% following chitosan coating. The authors attributed this decrease to interactions between the chitosan layer and the phospholipid bilayers, particularly with apolar head groups on the liposomal surface [43].

Drug loading capacity, which represents the amount of drug loaded per unit mass of particles [67], was significantly enhanced by chitosan coating. The drug loading capacities of L1-NDL and L2-NDL formulations were measured as 44.02 ± 2.15% and 45.65 ± 4.87%, respectively. Upon chitosan coating, these values increased to 61.47 ± 2.03% and 67.80 ± 0.74% for L1C-NDL and L2C-NDL formulations, respectively. Graphics of encapsulation efficiency and loading studies results are shown in Figs. (**1** and **2**), respectively.

### 3.3 Enzymatic Degradation Studies

In the enzymatic degradation studies, the primary objective was to assess the structural integrity and stability of the liposomal carrier in simulated gastric and intestinal environments. Therefore, instead of directly quantifying the drug content, the focus was placed on evaluating the degradation of the liposomal system as an indirect measure of formulation stability. This approach has been used in previous studies where the main aim was to investigate the behavior of the delivery system under enzymatic stress rather than tracking drug release kinetics [12, 68].

Conventional liposomes face significant stability challenges when exposed to the dynamic and harsh conditions of the gastrointestinal tract [25]. One of the main objectives in the development of oral liposome formulations is to enhance their stability against these conditions. Liposomes are prone to aggregation and degradation due to exposure to varying pH levels, pancreatic enzymes, and bile salts in the GI environment [60]. In this study, chitosan coating was employed to protect liposomes and improve their stability under such conditions.

**Fig. (3):**
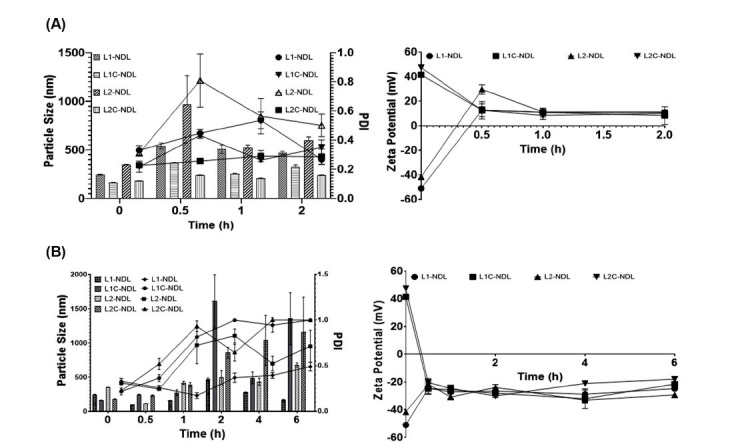
Formulations particle size, Polydispersity Index (PDI), and zeta potential change graph in media containing pepsin (Mean ± SD). (**A**), Formulations particle size, Polydispersity Index (PDI), and zeta potential change graph in media containing pancreatin (Mean ± SD). (**B**).

The particle size of chitosan-coated liposomes exhibited less variation in gastric media containing pepsin compared to uncoated liposomes. Among all formulations, L2C-NDL showed the least change in particle size and polydispersity index under these conditions. However, all formulations demonstrated an increase in particle size compared to their baseline values (Fig. **3A**).

The zeta potential changes of coated liposomes in gastric media were less pronounced compared to uncoated liposomes (Fig. **3A**). It is hypothesized that the negative charge arising from the phosphate groups of lipids and dicetyl phosphate in uncoated liposomes causes the adsorption of positively charged ions, leading to instability. This instability contributes to further changes in particle size and PDI values. Chitosan coating was shown to improve the stability of liposomes in the gastric environment [43].

In pancreatin-containing media, the particle size variation was more pronounced in chitosan-coated liposomes than in uncoated ones. The size of the chitosan-coated liposomes from the pancreatin-containing medium varied more than the uncoated ones. After 6 hours, the particle size of the formulations increased, except for L1-NDL. The results are given in Fig. (**3B**).

In simulated intestinal fluid, a significant increase in the particle size of coated liposomes was observed. This may be attributed to the reduced electrostatic interaction between chitosan and the liposome surface due to a decrease in the cationic charge of chitosan (Fig. **3B**). As these interactions weaken, the medium penetrates the particles, causing an increase in their size. Furthermore, pancreatic phospholipases exhibit a digestive effect on the phospholipids, which may contribute to the instability of particle size and PDI values [43, 69].

### 3.4 *In Vitro* Release Studies

**Fig. (4):**
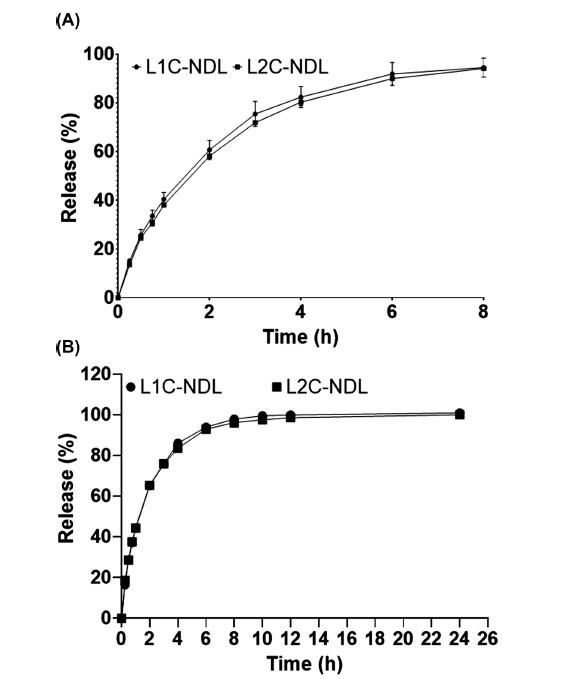
L1C-NDL and L2C-NDL release (%) of formulations in the gastric environment (± SD) (**A**), L1C-NDL and L2C-NDL release (%) of formulations in the intestinal environment (± SD) (**B**).

An *in vitro* release study was performed with L1C-NDL and L2C-NDL formulations at pH 1.2 and pH 6.8 using a dialysis membrane. During the gastric emptying phase (4 hours), L1C-NDL and L2C-NDL formulations showed 82.38% and 80.30% release, respectively. After 8 hours, both formulations exhibited approximately 94% of the release (Fig. **4A**). At the end of the intestinal emptying time (8 hours), L1C-NDL and L2C-NDL formulations demonstrated 97.91% and 96.19% release, respectively. By the 24-hour mark, both formulations achieved 100% release (Fig. **4B**).

Drug release has been evaluated separately in both the gastric and intestinal environments. Upon comparison of the drug release profiles in both media, it was observed that the drug was released at a slower rate in the gastric environment than in the intestinal environment. At the acidic pH of the gastric medium, the amino groups of chitosan become protonated, which enhances electrostatic interactions with the negatively charged phospholipids on the liposome surface. This interaction helps protect the liposomal contents under acidic conditions. However, in the intestinal environment with a higher pH, the interaction between chitosan and the liposome surface is reduced, leading to increased leakage and a faster release rate. In addition to all these, when the particle size and PDI values of chitosan-coated formulations were evaluated in enzymatic degradation studies, it was observed that chitosan-coated formulations were more stable in the gastric environment than in the intestinal environment. This result is consistent with *in vitro* release studies.

Similar results were obtained in the study by Sağıroğlu, where chitosan-coated liposomes loaded with carbamazepine and coenzyme Q10 were prepared, and *in vitro* release studies were conducted at pH 1.2 and 7.4. It was reported that liposomes released their contents more rapidly at the higher pH of 7.4, which is consistent with the findings of this study [70]. Furthermore, Nguyen *et al*. developed chitosan-coated nanoliposomes loaded with berberine hydrochloride for oral delivery. In their *in vitro* release study, they reported a higher release rate of berberine hydrochloride in simulated intestinal fluid compared to simulated gastric fluid, similar to the results observed in this study. Additionally, Nguyen *et al*. highlighted that slower release in the gastric environment is desirable for oral drug delivery, as it allows more drug to be released in the intestinal environment [43].

### 3.5 Permeability Studies

Although NDL has a well-documented safety profile, the current study involves a novel drug delivery system-liposomes-which may alter the interaction of the drug with biological membranes. Therefore, it was important to evaluate the cytotoxic potential of the liposomal formulation itself. Caco-2 cells were used to assess not only the cytotoxicity of the drug but also any potential cytotoxic effects arising from the liposomal carrier system.

The liposome formulation was composed of a mixture of soybean phospholipids, CH, DCP, and CO. Soybean lecithin, compared to animal-derived egg yolk lecithin, allows liposomes with higher permeability, making large-scale production more feasible. Studies in the literature suggest that lecithin derived from soybeans is more economical, safer, and more stable in production [71]. Additionally, lecithin is classified as Generally Recognized as Safe (GRAS) by regulatory authorities [72]. Chitosan is a crucial component of eukaryotic cell membranes. It influences the rigidity, thickness, and stability of the liposome membrane. An appropriate phospholipid-to-CH ratio enables controlled drug release from the carrier system [73]. DCP has been used to create negatively charged liposomes. The negative charge enhances the electrostatic affinity of the liposome to the positively charged cell membrane, thereby increasing the stability of the liposome [74].

A cell viability study was carried out to understand the toxic effects of the designed drug delivery system components and the active substance on intestinal cells in oral use. Cell viability was checked through the MTT test. Cell viability was over 95% in optimum chitosan-coated formulations. Based on these results, it is thought that the formulations do not have a cytotoxic effect [75]. Cell viability (%) rates are given in Fig. (**5**). The IC_50_ values of formulations were calculated by using the GraphPad Prism Program. According to results, while the IC_50_ value for L1C-NDL was found to be 1.23 ± 0.087 µg/mL, the IC_50_ value of L2C-NDL was found to be 1.56 ± 0.055 µg/mL.

In the permeability study, Caco-2 cells, which are colon cancer cells, were used, and the transition tracking was performed with HPLC. Transition direction and ratios of NDL are presented in Fig. (**6**).

Caco-2 cells were used to simulate the permeability of intestinal cells. Features such as the membrane, tight junctions, and active transport proteins are similar to those of intestinal cells [76]. Cell permeability of NDL-loaded liposome formulations was investigated from apical to basolateral (A→B) and basolateral to apical direction (B→A). The formulations showed higher permeability in the A→B direction. While the L1C-NDL formulation showed higher permeability in the first 30 minutes in the A→B direction, it showed almost equal permeability in both formulations after 2 hours. Although the formulations in the B→A direction showed varying permeability for 2 hours, the L1C-NDL formulation showed higher permeability.

TEER was measured before and after the experiment to assess cell integrity. Although the TEER values decreased after the experiment, it was not assumed that the cells were damaged. Because it has been reported in the literature that damage occurs with changes exceeding 40% [77]. The decrease in TEER value may be due to the effect of chitosan opening the tight junction areas of cells, which increases paracellular transport. The TEER value results of the study are provided in Fig. (**7**).

Xu *et al*. developed chitosan-coated liposomes to improve the oral bioavailability of emodin. These liposomes were prepared using a mixture of soybean lecithin, CH, and vitamin E. The transepithelial transport efficiency of the formulated liposomes was assessed using a Caco-2 cell monolayer model. In permeability studies, chitosan-coated emodin-loaded liposomes were compared with free emodin. The results showed a 4.7-fold increase in the apparent permeability coefficient (P_app_) value for the liposomal formulation. The authors concluded that chitosan-coated liposomes have the potential to significantly enhance the transepithelial transport of emodin across the Caco-2 cell monolayer [78]. Although the current findings are promising, further *in vivo* pharmacokinetic and efficacy studies would be beneficial to support the *in vitro* results and to provide deeper insights into the biodistribution, metabolism, and therapeutic potential of the developed system.

### 3.6 Stability Studies

Particle size, PDI, and zeta potential are critical parameters in evaluating the stability and functionality of nanostructured formulations. Changes in these parameters over time can influence the physical stability and efficacy of the formulation. In this study, the long-term stability of two formulations, L1C-NDL and L2C-NDL, was assessed, focusing on their particle size, PDI, zeta potential, mobility, and conductivity over 12 months (Table **4**). The initial particle size of L1C-NDL was 216.633 ± 4.720 nm, which decreased to 180.533 ± 1.482 nm by the 12th month. Similarly, the particle size of L2C-NDL decreased from 225.267 ± 4.645 nm to 173.700 ± 1.122 nm over the same period. No significant changes were observed in the particle size of L1C-NDL. For L1C-NDL, the PDI decreased from 0.264 ± 0.025 to 0.208 ± 0.060, while for L2C-NDL, the PDI dropped significantly from 0.357 ± 0.054 to 0.179 ± 0.043. The PDI values also showed a decreasing trend, indicating improved particle size distribution homogeneity [79]. These results confirm that the particle size distribution remained within the acceptable range [80]. The zeta potential of L1C-NDL exhibited a significant decrease, from 58.367 ± 1.066 mV to 23.767 ± 0.736 mV. In contrast, L2C-NDL showed a smaller reduction, from 39.067 ± 0.386 mV to 35.667 ± 0.411 mV. This is notable as literature suggests that lower zeta potential values can increase particle interactions, potentially leading to aggregation and larger particle sizes. The decrease in zeta potential could be attributed to a reduction in the surface electric field magnitude, a factor often associated with increased particle interactions. However, the absence of size increase demonstrates that the formulations retained their physical stability [81]. Electrophoretic mobility is a parameter related to zeta potential [82, 83]. A significant decrease in electrophoretic mobility was observed in L1C-NDL, while L2C-NDL showed only a slight reduction. Conductivity measurements indicated significant changes during the first month but stabilized in subsequent months. No significant changes were observed in the drug content quantification (Table **5**).

## CONCLUSION

Hypertension, a widespread chronic condition, is commonly managed through oral administration due to its convenience and patient compliance, especially for long-term therapy. In this study, liposomal formulations of Nadolol (NDL), an FDA-approved antihypertensive drug, were successfully developed as a potential alternative to conventional oral tablets. By employing chitosan (CH) coating, the liposomes were designed to withstand the harsh conditions of the gastrointestinal tract and to prolong mucosal residence time, thereby enhancing oral drug delivery performance. Comprehensive evaluations, including physicochemical characterization, encapsulation, loading efficiency, enzymatic degradation, *in vitro* release, and permeability studies, were conducted to assess the performance of the formulations. The optimized liposomes exhibited favorable release profiles and permeability characteristics, with no cytotoxic effects observed *in vitro*. These findings suggest that chitosan-coated liposomes offer a promising and effective platform for improving the stability, mucosal adhesion, and oral bioavailability of antihypertensive agents, such as NDL. Further *in vivo* studies are warranted to validate these outcomes and support potential clinical applications.

## Figures and Tables

**Fig. (1):**
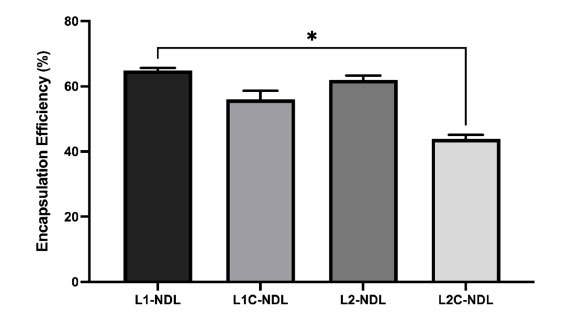
Encapsulation efficiency of liposome formulations. Statistically significant difference; * *p*<0.05; L1-NDL *vs*. L2C-NDL.

**Fig. (2):**
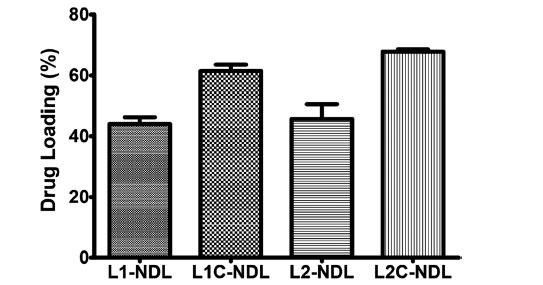
Drug loading capacity of liposome formulations (Mean ± SD).

**Fig. (5):**
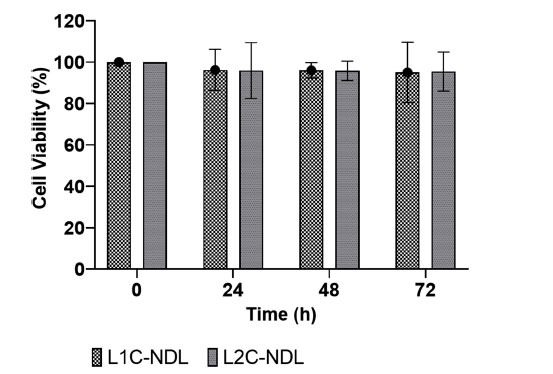
Cell viability (%) graph of optimum formulations containing NDL.

**Fig. (6):**
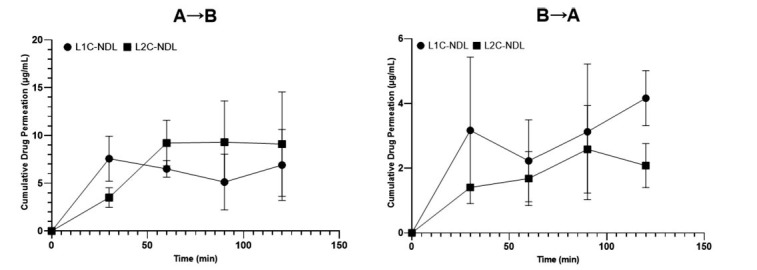
Data of NDL transition from apical to basolateral (A→B) and basolateral to apical (B→A) (Mean ± SD).

**Fig. (7):**
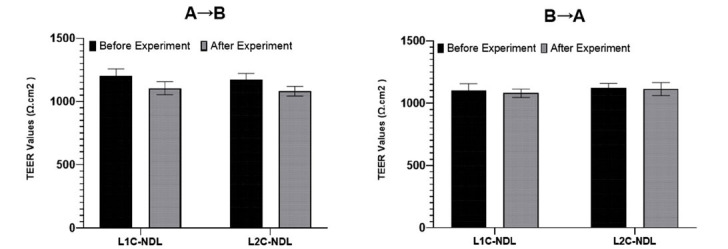
Graph of TEER values measured before and after the experiment in studies of NDL's transition from apical to basolateral (A→B) and basolateral to apical (B→A) (Mean ± SD).

**Table 1:** L1 and L2 formulation component ratios.

**Formulation**	**SPC (mg/mL)**	**DCP (mg/mL)**	**CH (mg/mL)**	**CO (mg/mL)**
L1	457	81.9	10	50
L2	457	81.9	0.032	-

**Table 2:** Particle size and PDI results of blank liposomes and chitosan-coated liposomes at varying concentrations.

**Formulations**	**Concentration (*w/w*)**	**Particle Size (nm)**	**PDI**	**Zeta Potential (mV)**
L1	0.4	241.50 ± 2.45	0.41 ± 0.01	53.00 ± 1.95
	0.3	186.40 ± 1.50	0.26 ± 0.01	53.40 ± 1.75
	0.25	163.30 ± 4.82	0.22 ± 0.04	41.40 ± 1.96
	0.2	161.00 ± 2.91	0.24 ± 0.02	43.50 ± 2.15
	0.1	677.20 ± 126.50	0.68 ± 0.10	24.90 ± 2.15
	0	246.60 ± 5.09	0.33 ± 0.03	-51.10 ± 1.13
L2	0.4	273.20 ± 35.48	0.36 ± 0.08	54.90 ± 0.85
	0.3	267.80 ± 47.37	0.40 ± 0.01	47.90 ± 1.21
	0.25	175.90 ± 3.85	0.23 ± 0.01	47.40 ± 1.06
	0.2	159.90 ± 4.57	0.27 ± 0.01	38.60 ± 2.31
	0.1	166.50 ± 4.25	0.27 ± 0.01	-41.80 ± 0.15
	0	350.90 ± 2.95	0.31 ± 0.03	-41.70 ± 1.64

**Table 3:** Particle size, polydispersity index, and zeta potential of formulations.

**Formulation Codes**	**Particle Size (nm)**	**PDI**	**Zeta Potential (mV)**
L1	246.60 ± 5.09	0.33 ± 0.03	-51.10 ± 1.13
L2	350.90 ± 2.95	0.31 ± 0.03	-41.40 ± 1.64
L1-NDL	27.02 ± 0.18	0.39 ± 0.01	-42.09 ± 0.29
L2-NDL	24.55 ± 0.22	0.37 ± 0.01	-50.91 ± 0.75
L1C	163.30 ± 4.82	0.22 ± 0.04	41.40 ± 1.96
L2C	175.90 ± 3.85	0.23 ± 0.01	47.40 ± 1.06
L1C-NDL	160.10 ± 3.17	0.19 ± 0.01	40.77 ± 2.17
L2C-NDL	161.00 ± 2.30	0.18 ± 0.02	37.84 ± 5.51

**Table 4:** Particle size, PDI, and zeta potential analysis of L1C-NDL and L2C-NDL formulations over a 12-month period.

**L1C-NDL**					
**Time (Months)**	**Particle Size (nm)**	**PDI**	**Zeta Potential (mV)**	**Mobility (µmcm/Vs)**	**Conductivity (mS/cm)**
**0**	216.633 ± 4.720	0.264 ± 0.025	58.367 ± 1.066	4.576 ± 0.084	0.606 ± 0.020
**1**	183.800 ± 2.535	0.211 ± 0.016	43.933 ± 0.556	3.445 ± 0.044	0.080 ± 0.001
**3**	222.133 ± 3.027	0.321 ± 0.019	38.833 ± 0.838	3.044 ± 0.066	0.082 ± 0.001
**6**	176.567 ± 2.042	0.195 ± 0.023	33.867 ± 1.181	2.655 ± 0.091	0.085 ± 0.002
**9**	181.267 ± 3.682	0.180 ± 0.037	28.433 ± 0.873	2.230 ± 0.068	0.086 ± 0.001
**12**	180.533 ± 1.482	0.208 ± 0.060	23.767 ± 0.736	1.864 ± 0.057	0.086 ± 0.000
**L2C-NDL**					
**Time (Months)**	**Particle Size (nm)**	**PDI**	**Zeta Potential (mV)**	**Mobility (µmcm/Vs)**	**Conductivity (mS/cm)**
**0**	225.267 ± 4.645	0.357 ± 0.054	39.067 ± 0.386	3.063 ± 0.032	0.155 ± 0.000
**1**	179.167 ± 0.943	0.150 ± 0.012	37.600 ± 0.535	2.947 ± 0.041	0.06 ± 0.000
**3**	178.733 ± 1.438	0.092 ± 0.031	38.033 ± 1.034	2.979 ± 0.081	0.063 ± 0.002
**6**	176.367 ± 3.608	0.169 ± 0.000	37.000 ± 0.432	2.901 ± 0.037	0.062 ± 0.000
**9**	179.033 ± 1.247	0.147 ± 0.033	37.133 ± 0.850	2.912 ± 0.065	0.063 ± 0.000
**12**	173.700 ± 1.122	0.179 ± 0.043	35.667 ± 0.411	2.798 ± 0.032	0.063 ± 0.000

**Table 5:** Results of % NDL measurements in the stability study of L1C-NDL and L2C-NDL formulations.

**Time (Months)**	**L1C-NDL**			**L2C-NDL**		
	**5** ± 3°**C**	**25** ± 2°**C**	**40** ± 2°**C**	**5** ± 3°**C**	**25** ± 2°**C**	**40** ± 2°**C**
**0**	100.120 ± 0.352	100.001 ± 0.520	100.210 ± 0.551	100.325 ± 1.200	100.520 ± 0.764	100.408 ± 0.585
**3**	95.580 ± 1.674	91.998 ± 0.046	90.900 ± 0.124	92.265 ± 0.313	91.053 ± 0.034	89.420 ± 0.030
**6**	94.964 ± 0.922	91.738 ± 0.031	90.223 ± 0.676	92.074 ± 0.818	90.621 ± 0.081	89.097 ± 0.043
**12**	94.111 ± 0.032*	91.436 ± 0.008*	89.469 ± 0.064*	91.309 ± 0.078*	90.554 ± 0.022*	88.981 ± 0.032*

**Note**: Statistically significant difference; *p<0.05 *vs*. Day 0. Data are shown as Mean ± S.D.

## Data Availability

All data generated or analyzed during this study are included in this published article.

## LIST OF ABBREVIATIONS

A→B: Apical to Basolateral
B→A: Basolateral to Apical
Caco-2: Human Colon Epithelial Cancer Cell Line
CH: Cholesterol
CO: Cinnamon Oil
DCP: Dicetyl Phosphate
DMEM: Dulbecco's Modified Eagle Media
FDA: Food and Drug Administration
HPLC: High Performance Liquid Chromatography
MTT: 3-[4,5-Dimethylthiazol-2-yl]-2,5-Diphenyltetra-zolium Bromid
NDL: Nadolol
PDI: Polydispersity Index
SPC: L-α-phosphatidylcholine
TEER: Transepithelial Electrical Resistance

## References

[1] Miyazaki N., Misaka S., Ogata H., Fukushima T., Kimura J. (2013). Effects of itraconazole, dexamethasone and naringin on the pharmacokinetics of nadolol in rats.. Drug Metab. Pharmacokinet..

[2] Fumagalli C., Maurizi N., Marchionni N., Fornasari D. (2020). β-blockers: Their new life from hypertension to cancer and migraine.. Pharmacol. Res..

[3] Kalsoom S., Zamir A., Rehman A. (2022). Clinical pharmacokinetics of nadolol: A systematic review.. J. Clin. Pharm. Ther..

[4] Priviero F. (2023). Epigenetic modifications and fetal programming: Molecular mechanisms to control hypertension inheritance.. Biochem. Pharmacol..

[5] Mills K.T., Stefanescu A., He J. (2020). The global epidemiology of hypertension.. Nat. Rev. Nephrol..

[6] Adeyeye E., Kapil V., Lobo M.D. (2022). Hypertension.. Medicine.

[7] O’Shea P.M., Griffin T.P., Fitzgibbon M. (2017). Hypertension: The role of biochemistry in the diagnosis and management.. Clin. Chim. Acta.

[8] Ammann E.M., O’Brien E.S., Milentijevic D. (2023). Characteristics, management, and blood pressure control in patients with apparent resistant hypertension in the US.. Heliyon.

[9] Byrd J.B., Ram C.V.S., Lerma E.V., Lerma E.V., Sparks M.A., Topf J.M. (2019). Pharmacologic treatment of hypertension.. Nephrol Secrets..

[10] Volkova T.V., Simonova O.R., Vigurskaya T.A., Perlovich G.L. (2023). Thermodynamics of solubility, distribution and permeability processes exemplified by nadolol - A beta-blocker drug with antianxiety potential.. J. Mol. Liq..

[11] Kawabata Y., Wada K., Nakatani M., Yamada S., Onoue S. (2011). Formulation design for poorly water-soluble drugs based on biopharmaceutics classification system: Basic approaches and practical applications.. Int. J. Pharm..

[12] Mozafari M.R. (2005). Liposomes: An overview of manufacturing techniques.. Cell. Mol. Biol. Lett..

[13] Sercombe L., Veerati T., Moheimani F., Wu S.Y., Sood A.K., Hua S. (2015). Advances and challenges of liposome assisted drug delivery.. Front. Pharmacol..

[14] Nsairat H., Alshaer W., Odeh F., Esawi E., Khater D., Al Bawab A. (2023). Recent advances in using liposomes for delivery of nucleic acid-based therapeutics.. OpenNano.

[15] Shukla R., Handa M., Vasdev N., Singh D.P., Kesharwani P., Kesharwani P., Taurin S., Greish K. (2021). Nanomedicine in pain management.. Theory Applications of Nonparenteral Nanomedicines..

[16] Nsairat H., Khater D., Sayed U., Odeh F., Al Bawab A., Alshaer W. (2022). Liposomes: Structure, composition, types, and clinical applications.. Heliyon.

[17] Quadir S.S., Saharan V., Choudhary D., Harish, Jain C.P., Joshi G. (2022). Nano-strategies as oral drug delivery platforms for treatment of cancer: Challenges and future perspectives.. AAPS PharmSciTech.

[18] Noble G.T., Stefanick J.F., Ashley J.D., Kiziltepe T., Bilgicer B. (2014). Ligand-targeted liposome design: Challenges and fundamental considerations.. Trends Biotechnol..

[19] Liu P., Chen G., Zhang J. (2022). A Review of liposomes as a drug delivery system: Current status of approved products, regulatory environments, and future perspectives.. Molecules.

[20] Lou J., Duan H., Qin Q. (2023). Advances in oral drug delivery systems: Challenges and opportunities.. Pharmaceutics.

[21] Bozzuto G., Molinari A. (2015). Liposomes as nanomedical devices.. Int. J. Nanomedicine.

[22] Bruch G.E., Fernandes L.F., Bassi B.L.T. (2019). Liposomes for drug delivery in stroke.. Brain Res. Bull..

[23] Large D.E., Abdelmessih R.G., Fink E.A., Auguste D.T. (2021). Liposome composition in drug delivery design, synthesis, characterization, and clinical application.. Adv. Drug Deliv. Rev..

[24] Woodley J.F. (1994). Enzymatic barriers for GI peptide and protein delivery.. Crit. Rev. Ther. Drug Carrier Syst..

[25] He H., Lu Y., Qi J., Zhu Q., Chen Z., Wu W. (2019). Adapting liposomes for oral drug delivery.. Acta Pharm. Sin. B.

[26] Yi X., Chen Y., Gao X., Gao S., Xia G., Shen X. (2025). Enhancement of digestive stability in curcumin-loaded liposomes *via* glycolipids: An analysis *in vitro* and in vivo.. Food Res. Int..

[27] Bernkop-Schnürch A., Dünnhaupt S. (2012). Chitosan-based drug delivery systems.. Eur. J. Pharm. Biopharm..

[28] Picos-Corrales L.A., Morales-Burgos A.M., Ruelas-Leyva J.P. (2023). Chitosan as an outstanding polysaccharide ımproving health-commodities of humans and environmental protection.. Polymers.

[29] Zhang T., Yu H., Li C. (2025). Study of the interaction between polysaccharides and liposomes based on low-field nuclear magnetic resonance.. Food Biosci..

[30] Elkomy M.H., Ali A.A., Eid H.M. (2022). Chitosan on the surface of nanoparticles for enhanced drug delivery: A comprehensive review.. J. Control. Release.

[31] Wu W., Lu Y., Qi J. (2015). Oral delivery of liposomes.. Ther. Deliv..

[32] Alghareeb S., Ekenna I., Asare-Addo K., Conway B.R., Adebisi A.O. (2025). Chitosan nanoparticles for nasal drug delivery.. J. Drug Deliv. Sci. Technol..

[33] Kumar A., Yadav S., Pramanik J. (2023). Chitosan-based composites: Development and perspective in food preservation and biomedical applications.. Polymers.

[34] Patel V., Prajapati B., Patel M. (2007). Design and characterization of chitosan-containing mucoadhesive buccal patches of propranolol hydrochloride.. Acta Pharm..

[35] Das U., Kapoor D.U., Singh S., Prajapati B.G. (2024). Unveiling the potential of chitosan-coated lipid nanoparticles in drug delivery for management of critical illness: A review.. Z. Naturforsch. C J. Biosci..

[36] Alomrani A., Badran M., Harisa G.I. (2019). The use of chitosan-coated flexible liposomes as a remarkable carrier to enhance the antitumor efficacy of 5-fluorouracil against colorectal cancer.. Saudi Pharm. J..

[37] Erdem S., Türkoǧlu M. (2010). Glycyl-l-histidyl-l-liysine-Cu(2) loaded liposome formulations.. Marmara Pharm. J..

[38] Hosseini S.M., Abdouss M., Mazinani S., Soltanabadi A., Kalaee M. (2022). Modified nanofiber containing chitosan and graphene oxide-magnetite nanoparticles as effective materials for smart wound dressing.. Compos., Part B Eng..

[39] Zhou F., Xu T., Zhao Y. (2018). Chitosan-coated liposomes as delivery systems for improving the stability and oral bioavailability of acteoside.. Food Hydrocoll..

[40] (2003). Nadolol.. https://www.waters.com/nextgen/es/es/library/application-notes/2003/nadolol.html?srsltid=AfmBOoq3uaqHdRwkW0mfcA_x6_5v3EQmd0cdM-LhuESIx_5nFFUcjVkR.

[41] Gradauer K., Barthelmes J., Vonach C. (2013). Liposomes coated with thiolated chitosan enhance oral peptide delivery to rats.. J. Control. Release.

[42] Üstündağ Okur N., Yurdasiper A., Gündoğdu E., Homan G.E. (2016). Modification of solid lipid nanoparticles loaded with nebivolol hydrochloride for improvement of oral bioavailability in treatment of hypertension: Polyethylene glycol versus chitosan oligosaccharide lactate.. J. Microencapsul..

[43] Nguyen T.X., Huang L., Liu L., Elamin Abdalla A.M., Gauthier M., Yang G. (2014). Chitosan-coated nano-liposomes for the oral delivery of berberine hydrochloride.. J. Mater. Chem. B Mater. Biol. Med..

[44] İlem-Özdemir D., Gündoğdu E., Ekinci M., Aşıkoğlu M. (2016). Radioactive permeability studies of doxycycline hyclate from microemulsion and solution.. Marmara Pharm. J..

[45] Erkekoğlu P., Baydar T. (2021). Güncel *in vitro* sitotoksisite testleri.. Hacettepe Univ J Fac Pharm.

[46] Shariare M.H., Rahman M., Lubna S.R. (2020). Liposomal drug delivery of Aphanamixis polystachya leaf extracts and its neurobehavioral activity in mice model.. Sci. Rep..

[47] Nazlı H., Mesut B., Özsoy Y. (2021). Pharmaceutical approaches for low solubility agents and solubility of aprepitant.. J. Pharm. Sci..

[48] Çoban Ö., Değim Z. (2018). Development of Nanocochleates containing erlotinib HCl and dexketoprofen trometamol and evaluation of ın vitro characteristic properties.. Turk J Pharm Sci.

[49] Çoban Ö., Değim Z., Yılmaz Ş., Altıntaş L., Arsoy T., Sözmen M. (2019). Efficacy of targeted liposomes and nanocochleates containing imatinib plus dexketoprofen against fibrosarcoma.. Drug Dev. Res..

[50] Guimarães D., Cavaco-Paulo A., Nogueira E. (2021). Design of liposomes as drug delivery system for therapeutic applications.. Int. J. Pharm..

[51] Channarong S., Chaicumpa W., Sinchaipanid N., Mitrevej A. (2011). Development and evaluation of chitosan-coated liposomes for oral DNA vaccine: The improvement of Peyer’s patch targeting using a polyplex-loaded liposomes.. AAPS PharmSciTech.

[52] Nguyen V.H., Thuy V.N., Van T.V., Dao A.H., Lee B.J. (2022). Nanostructured lipid carriers and their potential applications for versatile drug delivery *via* oral administration.. OpenNano.

[53] Rao J., McClements D.J. (2011). Formation of flavor oil microemulsions, nanoemulsions and emulsions: Influence of composition and preparation method.. J. Agric. Food Chem..

[54] Li X., Tang C., Salama M. (2022). Encapsulation efficiency and oral delivery stability of chitosan–liposome‐encapsulated immunoglobulin Y.. J. Food Sci..

[55] Grigoras A.G. (2017). Polymer-lipid hybrid systems used as carriers for insulin delivery.. Nanomedicine.

[56] Sebaaly C., Trifan A., Sieniawska E., Greige-Gerges H. (2021). Chitosan-coating effect on the characteristics of liposomes: A focus on bioactive compounds and essential oils: A review.. Processes.

[57] Das A., Ghosh S., Pramanik N. (2024). Chitosan biopolymer and its composites: Processing, properties and applications- A comprehensive review.. Hybrid Advances.

[58] Han J., Meade J., Devine D., Sadeghpour A., Rappolt M., Goycoolea F.M. (2024). Chitosan-coated liposomal systems for delivery of antibacterial peptide LL17-32 to Porphyromonas gingivalis.. Heliyon.

[59] Ahad A., Raish M., Bin Jardan Y.A., Al-Mohizea A.M., Al-Jenoobi F.I. (2024). Chitosan-tethered liposomes for sinapic acid delivery.. J. Drug Deliv. Sci. Technol..

[60] Liu Y., Yang T., Wei S. (2018). Mucus adhesion- and penetration-enhanced liposomes for paclitaxel oral delivery.. Int. J. Pharm..

[61] Andra V.V.S.N.L., Pammi S.V.N., Bhatraju L.V.K.P., Ruddaraju L.K. (2022). A Comprehensive review on novel liposomal methodologies, commercial formulations, clinical trials and patents.. Bionanoscience.

[62] Alavi S., Haeri A., Dadashzadeh S. (2017). Utilization of chitosan-caged liposomes to push the boundaries of therapeutic delivery.. Carbohydr. Polym..

[63] Wróblewska A.M., Samsonowicz-Górski J., Kamińska E., Drozd M., Matczuk M. (2022). Optimization of a CE-ICP-MS/MS method for the investigation of liposome–cisplatin nanosystems and their interactions with transferrin.. J. Anal. At. Spectrom..

[64] Gopi S., Balakrishnan P. (2021). Evaluation and clinical comparison studies on liposomal and non-liposomal ascorbic acid (vitamin C) and their enhanced bioavailability.. J. Liposome Res..

[65] Li K., Zhong W., Li P., Ren J., Jiang K., Wu W. (2023). Antibacterial mechanism of lignin and lignin-based antimicrobial materials in different fields.. Int. J. Biol. Macromol..

[66] Siva S., Jin J.O., Choi I., Kim M. (2023). Nanoliposome based biosensors for probing mycotoxins and their applications for food: A review.. Biosens. Bioelectron..

[67] Yin Y.M., Cui F.D., Mu C.F. (2009). Docetaxel microemulsion for enhanced oral bioavailability: Preparation and *in vitro* and *in vivo* evaluation.. J. Control. Release.

[68] Immordino M.L., Dosio F., Cattel L. (2006). Stealth liposomes: Review of the basic science, rationale, and clinical applications, existing and potential.. Int. J. Nanomedicine.

[69] Chen D., Xia D., Li X. (2013). Comparative study of Pluronic® F127-modified liposomes and chitosan-modified liposomes for mucus penetration and oral absorption of cyclosporine A in rats.. Int. J. Pharm..

[70] Sağıroğlu A.A. (2021). Chitosan-coated liposome-containing carbamazepine and coenzyme Q10: Design, optimization and evaluation.. J. Liposome Res..

[71] Le N.T.T., Cao V.D., Nguyen T.N.Q., Le T.T.H., Tran T.T., Hoang Thi T.T. (2019). Soy lecithin-derived liposomal delivery systems: Surface modification and current applications.. Int. J. Mol. Sci..

[72] Milić J., Čalija B., Dordević S.M., Čalija B. (2017). Diversity and functionality of excipients for micro/nanosized drug carriers. microsized nanosized carriers.. Microsized and Nanosized Carriers for Nonsteroidal Anti-Inflammatory Drugs..

[73] Kaddah S., Khreich N., Kaddah F., Charcosset C., Greige-Gerges H. (2018). Cholesterol modulates the liposome membrane fluidity and permeability for a hydrophilic molecule.. Food Chem. Toxicol..

[74] Jeon H.S., Seo J.E., Kim M.S. (2013). A retinyl palmitate-loaded solid lipid nanoparticle system: Effect of surface modification with dicetyl phosphate on skin permeation *in vitro* and anti-wrinkle effect in vivo.. Int. J. Pharm..

[75] Bhat U.M., Khan N.A., Raza S.N. (2024). Ciprofloxacin hydrochloride-loaded ocular silk fibroin liposomes: Formulation, characterisation, *in vitro* cytotoxicity, and antimicrobial activity.. Heliyon.

[76] Cui M., Wu W., Hovgaard L., Lu Y., Chen D., Qi J. (2015). Liposomes containing cholesterol analogues of botanical origin as drug delivery systems to enhance the oral absorption of insulin.. Int. J. Pharm..

[77] Okur N.Ü., Çağlar E.Ş., Kaynak M.S., Diril M., Özcan S., Karasulu H.Y. (2024). Enhancing oral bioavailability of domperidone maleate: Formulation, *in vitro* permeability evaluation ın-Caco-2 cell monolayers and *in situ* ratıntestinal permeability studies.. Curr. Drug Deliv..

[78] Xu Z., Hou Y., Sun J. (2022). Deoxycholic acid-chitosan coated liposomes combined with *in situ* colonic gel enhances renal fibrosis therapy of emodin.. Phytomedicine.

[79] Danaei M., Dehghankhold M., Ataei S. (2018). Impact of particle size and polydispersity ındex on the clinical applications of lipidic nanocarrier systems.. Pharmaceutics.

[80] Qiang F., Shin H.J., Lee B.J., Han H.K. (2012). Enhanced systemic exposure of fexofenadine *via* the intranasal administration of chitosan-coated liposome.. Int. J. Pharm..

[81] Montes C., Villaseñor M.J., Ríos Á. (2019). Analytical control of nanodelivery lipid-based systems for encapsulation of nutraceuticals: Achievements and challenges.. Trends Food Sci. Technol..

[82] Mudalige T., Qu H., Van Haute D., Ansar S.M., Paredes A., Ingle T. (2019). Characterization of nanomaterials: Tools and challenges..

[83] Engelhardt M.B., Sugimoto T., Papastavrou G., Kobayashi M. (2024). Electrophoretic mobility of nanoparticle aggregates: Independence from aggregate size.. Colloids Surf. A Physicochem. Eng. Asp..

